# Prevalence of Urinary Incontinence in CrossFit Practitioners before and during the COVID-19 Quarantine and its Relationship with Training Level: An Observational Study

**DOI:** 10.1055/s-0041-1739463

**Published:** 2021-12-06

**Authors:** Maita Poli de Araujo, Luiz Gustavo Oliveira Brito, Alberto de Castro Pochini, Benno Ejnisman, Marair Gracio Ferreira Sartori, Manoel João Batista Castello Girão

**Affiliations:** 1 Department of Gynecology, Escola Paulista de Medicina, Universidade Federal de São Paulo, São Paulo, SP, Brazil; 2 Department of Gynecology and Obstetrics, University of Campinas, Campinas, SP, Brazil

**Keywords:** crossfit, cross-sectional study, urinary incontinence, COVID-19, quarantine, crossfit, estudo observacional, incontinência urinária, COVID-19, quarentena

## Abstract

**Objective** To compare the prevalence of urinary incontinence (UI) before and during the COVID-19 quarantine in CrossFit women and their relationship with training level.

**Methods** A cross-sectional study was performed among 197 women practicing CrossFit. The inclusion criteria were nulliparous women, between 18 and 45 years old, who had trained, before quarantine, in accredited gyms. The exclusion criteria were not following the COVID-19 prevention protocols and having UI on other occasions than just sport. An online questionnaire was emailed containing questions about frequency, duration, and intensity of training and data related to the COVID-19 pandemic. The participants were invited to answer whether they were infected with COVID-19 and what treatment/recommendation they have followed. Whether UI stopped among participants, they were asked about the possible reasons why this happened. The training intensity was categorized as “the same,” “decreased” or “increased.”

**Results** The mean age of the participants was 32 years old and most (98.5%) could practice CrossFit during the pandemic. There was a decrease in training intensity in 64% of the respondents. Exercises with their own body weight, such as air squat (98.2%), were the most performed. Urinary incontinence was reported by 32% of the participants before the COVID-19 pandemic, and by only 14% of them during the pandemic (odds ratio [OR] = 0.32 [0.19–0.53]; *p* < 0.01; univariate analysis). Practitioners reported that the reason possibly related to UI improvement was the reduction of training intensity and not performing doubleunder exercise.

**Conclusion** The reduction in the intensity of CrossFit training during the COVID-19 quarantine decreased the prevalence of UI among female athletes.

## Resumo

**Objetivo** Comparar a prevalência de incontinência urinária (IU) no CrossFit, antes e durante a quarentena por COVID-19, e sua relação com a intensidade do treinamento.

**Métodos** Estudo observacional com 197 atletas de CrossFit. Os critérios de inclusão foram: nulíparas, 18 a 45 anos, treinando antes da quarentena em academias credenciadas. Os critérios de exclusão foram: não seguir os protocolos de prevenção da COVID-19 e ter IU em outras ocasiões que não apenas no esporte. Utilizou-se um questionário online com perguntas sobre frequência, duração e intensidade do treinamento e dados relacionados à pandemia, além de caso tivessem tido infecção pelo SARS-COV2, qual tratamento/recomendação seguiram. Caso a IU tenha parado entre as participantes, elas foram perguntadas quanto quais as possíveis razões pelas quais isso aconteceu. A intensidade do treinamento foi categorizada como “igual,” “diminuída” ou “aumentada “.

**Resultados** A média de idade foi de 32 anos e a maioria (98,5%) conseguiu praticar CrossFit durante a pandemia. Houve uma diminuição na intensidade do treinamento em 64% das entrevistadas. Exercícios com o próprio peso corporal, como agachamento no ar (98,2%), foram os mais realizados. Incontinência urinária foi relatada por 32% das participantes antes da pandemia e por apenas 14% durante a pandemia (odds ratio [OR] = 0,32 [0,19–0,53]; *p* < 0,01). As atletas relataram que o motivo possivelmente relacionado à melhora da IU foi a redução da intensidade do treinamento e não realizar o exercício doubleunder.

**Conclusão** A redução da intensidade do treinamento de CrossFit durante a quarentena por COVID-19 diminuiu a prevalência de IU entre as atletas.

## Introduction

Urinary incontinence (UI) during exercise, also termed athletic incontinence, is defined as the involuntary loss of urine during physical exercise. 1 High-performance and high-impact sports, such as acrobatic trampoline, weightlifting, and long-distance running, may cause a 2-fold to increase in the odds for UI in young athletes. 2 3 In middle-aged women, losing urine significantly limits physical activity. 4 5

**Fig. 1:**
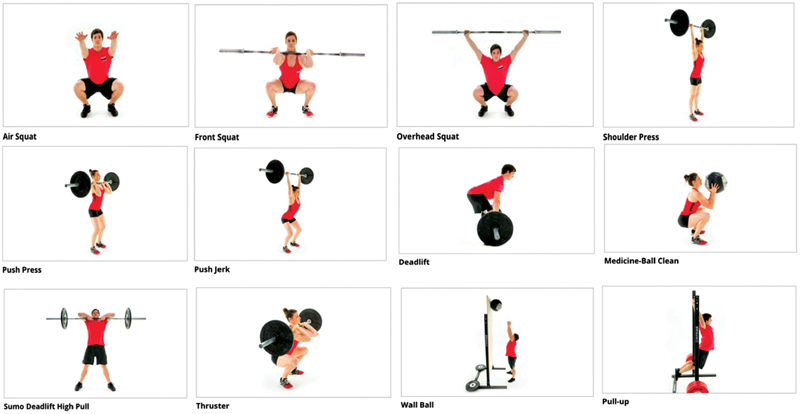
Basic elements of crossfit.

CrossFit is an example of high-intensity functional training (HIFT). The training consists of a combination of different exercise elements: endurance, gymnastics, and weightlifting exercises. CrossFit training sessions are organized into joint mobility, warm-up, technical part, and the main part. There are 12 different types of movements registered in CrossFit ( Fig. 1 ): air squat, front squat, overhead squat, shoulder press, push press, push jerk, deadlift, medicine-ball clean, sumo deadlift high pull, thruster, wall ball, and pull-up. 6

Recent studies have shown that the prevalence of UI in CrossFit practitioners is of ∼ 30%, with skipping rope the most related to this event. 6 7 The etiopathogenesis of this condition is related to the increase in intra-abdominal pressure that is not balanced by the contraction of the pelvic muscles. 8 In addition, the displacement of the pelvic organs during the skipping rope movement also precipitates urine loss. 9

The level of physical activity is intimately related with the occurrence of UI during sport. Standards such as frequency, intensity, and training level are important to assess incontinent athletes. In this regard, physical exercises for health (at least 30 minutes a day, with moderate intensity, 5 days a week) do not have a negative effect on the pelvic floor. 5 6

Due to the COVID-19 pandemic, the Ministry of Health counselled quarantine in March 2020 and ordered the closure of nonessential services, including CrossFit gyms. 10 Patients affected by the mild form of the disease were instructed to undergo social isolation, and the most severe cases were referred to the health service. 11 12 However, staying physically active during the COVID-19 pandemic was a recommendation by the World Health Organization (WHO) to maintain immunity. 9 10 Thus, it is not known how sportspeople have adapted their training during quarantine and whether modifying their sports schedule could impact UI. We consider that it is possible that social isolation and reduced training intensity may be beneficial for this population. Given that, we sought to compare the prevalence of UI before and during the quarantine by COVID-19 in CrossFit women and their relationship with training level.

## Methods

The present cross-sectional study was conducted immediately after the quarantine of Brazil following the guidelines of the Strengthening the Reporting of Observational Studies in Epidemiology Statement (STROBE) guidelines. The Institutional Review Board of the Universidade Federal de São Paulo, São Paulo, state of São Paulo Brazil, approved our study protocol (CAAE 31964920.1.0000.5505) and the respondents read and signed an informed consent before starting an online survey. Thus, after signing a yes-no question to confirm their willingness to participate voluntarily, they completed a questionnaire. Patients or the public were not involved in the design, conduct, reporting, or dissemination plans of our research.

The COVID-19 pandemic in Brazil was confirmed on 25 February 2020, when a man from São Paulo tested positive for the infection. On March 27 ^th^ , Brazil declared a temporary prohibition on foreign air travelers and most state governors-imposed quarantines to prevent the spread of the virus. Data collections were performed in April, May, and June 2020.

A Google Forms ( https://forms.gle/9HbTvZEAgTbxExvy7 ) questionnaire was sent to athletes registered on the site www.crosscontinencebrazil and who had already participated in a survey on athletic incontinence 6 . The inclusion criteria were nulliparous women between 18 and 45 years old who had trained in accredited CrossFit gyms before quarantine in Brazil ( maps.crossfit.com ), at least 30 minutes a day, 3 times a week. Moreover, as inclusion criteria, were considered female athletes that were following the pandemic protocols: maintaining social distance, staying at home most of the time, not attending gyms, and practicing social isolation in case of contamination by SARS-COV2. The exclusion criteria were not following the COVID-19 pandemic protocols and patients with UI on other occasions than just sports.

The questionnaire consisted of demographic variables (age, occupation, housing, and number of people living in the same household), variables related to CrossFit training (frequency, duration, and intensity), variables related to COVID-19 (severity of symptoms, type of treatment), and urinary incontinence. Participants were asked to answer whether they were infected with COVID-19 and what treatment/recommendation they had followed. These variables were categorized into social isolation (mild clinical manifestations) or hospital treatment (moderate to severe clinical manifestations).

To evaluate symptoms of UI before and during quarantine, participants were asked: “ *before quarantine, have you lost urine during CrossFit training?”* and *“during quarantine, have you lost urine during CrossFit training?”.* Possible answers were yes or no. Moreover, an open-ended question was included: “ *if you did not lose urine during exercise this quarantine, what was the main reason”.* The analysis of open-ended questions was performed by categorizing the results and grouping by similarity.

Sample size calculation was based on previous reports. 6 Thus, stipulating a 95% confidence interval (CI), a hypothesized prevalence of 50% reduction of physical activity and a standard error of 5%, a total sample of 169 subjects was established. The statistical treatment was descriptive and parametric tests were used for categorical variables, with a significance level of *p* < 0.05. Univariate analysis with odds ratio (OR) and 95%CIs were also calculated for some associations.

## Results

Table 1 depicts the general characteristics of the participants. A total of 197 female athletes were enrolled in the present study. The mean age of the participants was 32 years old, and most of them had an adequate body mass index (82.7%). Most participants lived in an apartment (65.5%), and most of them reported that they lived with another person (40.1%). Most athletes (98.5%) managed to train CrossFit during the pandemic.

**Table 1:** General characteristics of female CrossFit practitioners during the COVID-19 quarantine

Variable	*n*	%
Type of housing during the quarantine		
Home	68	34.5
Apartment	129	65.5
Number of people in the household		
Alone	26	13.2
2	79	40.1
3	54	27.4
> 3	38	19.3
Body Mass Index (Kg/m2)		
Underweight (< 18.5)	0	
Normal (18.5–24.9)	163	82.7
Overweight (25–29.9)	27	13.7
Obese (> 30)	7	3.6
Reported COVID-19 symptoms		
Yes	22	11.2
No	175	88.8
Type of treatment for COVID-19		
Social isolation (home)	22	100
Hospitalization	0	0
Were you able to train CrossFit during the quarantine?		
Yes	194	98.5
No	3	1.5

SARS-CoV 2infection manifested in a mild format in 22 athletes and treatment was performed conservatively.

Table 2 presents the data related to duration, frequency, intensity, and training level of CrossFit of these athletes that were able to be physically active during the pandemic. The main training site for CrossFit during the quarantine was at home (55%), followed by the outside area of the building or home (19.4%). While most athletes maintained a high frequency of training per week (> 5 days) with a duration > 30 minutes per day, training intensity reduced in 64%.

**Table 2:** Place, frequency, duration, and intensity of CrossFit training during the quarantine

Variables	*n*	%
Location where you trained during the quarantine		
Indoor house or apartment	108	55
On a small balcony in the house or apartment	26	13.4
Outside the house or apartment	58	19.4
In the garage of the house or apartment	2	1.9
Frequency of training/week		
3	28	14.4
4	22	11.3
5	48	24.7
6	43	22.2
7	53	27.3
Training duration (minutes) per day		
30	36	18.6
30–60	107	55.2
> 60	51	26.3
Intensity of CrossFit training during the quarantine		
Increased	0	0
Same	69	36
Decreased	126	64

**Fig. 2:**
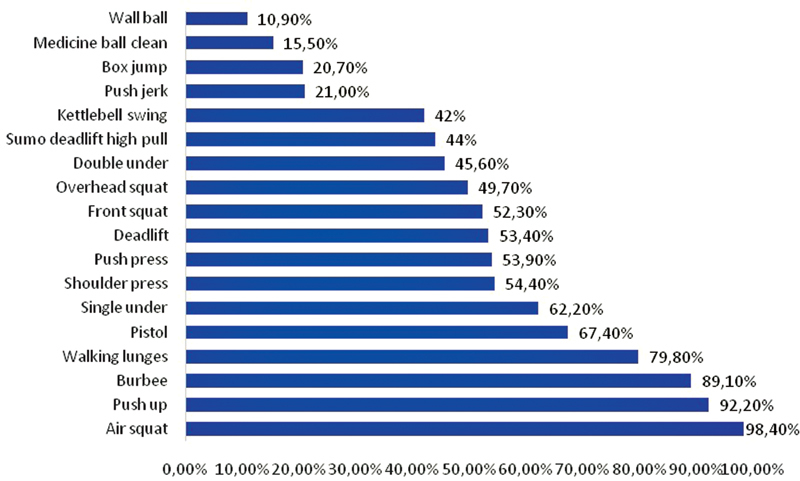
Types of CrossFit exercises performed at home during the COVID-19 pandemic.

Figure 2 displays the most prevalent exercises chosen by the respondents. In general, exercises using their own body weight, such as air squat, were the most performed (98.2%), followed by push up (92.2%) and burpee (89.1%). Exercises that needed specific materials, such as medicine ball and wall ball, were practiced by less than half of the athletes.

Regarding UI, before the quarantine, 32% of the athletes reported the symptom and, during the quarantine, only 14% of the participants reported UI. This reduction was statistically significant (OR = 0.32 [0.19–0.5307]; *p* < 0.001) by univariate analysis ( Table 3 ). When the athletes were asked what reasons they considered to be the cause for UI improvement ( *n* = 36), 22 reported they had reduced the training intensity and 14 stopped doing exercises with rope.

**Table 3:** Urinary incontinence before and after the quarantine

	Before the quarantine	During the quarantine	Total
With UI	63 (32%)	27 (14%)	90
Without UI	134 (68%)	170 (86%)	304
Marginal column totals	197	197	394

Abbreviation: UI, urinary incontinence.

z-statistic= 4.427; *p* <0.001; OR = 0.32 [0.19–0.53] – univariate analysis.

## Discussion

Our study has found a 64% reduction on CrossFit training during the quarantine and an 18% reduction of UI during this period. The sudden onset of a state of social isolation implies a radical change in the lifestyle of the population, such as the level of physical activity, eating habits, and sleep. 13 Thus, many medical societies released information on how to avoid a sedentary lifestyle during the COVID-19 pandemic. 14 15 At the same time, gyms and elite athletes provided free online training to help people stay active at home. 16 One of the main characteristics of CrossFit training is that it is performed in a gym equipped with rings, bars, and weights. 17 Therefore, one of the possibilities of the present study was that CrossFit practitioners would not be able to train at home. However, it was observed that 98.5% of the interviewees maintained their training, adapting their exercise routine indoors.

Exercises that needed special equipment, such as wall ball and medicine ball, were the least practiced by the participants (10.9 and 15.5%, respectively), while exercises using the own body weight of the participants, such as air squat and push up, were maintained by most athletes (98.4 and 98.2%, respectively). Another interesting fact is that the specificity of CrossFit training involves the association of Olympic lifting exercises (squats, sprints, throws); aerobic exercises (such as rowing, running, and cycling) and gymnastics movements (such as handstands, parallel bars, rings, and bars). 18 In this sense, it was a surprise to realize in our study that the interviewees were able to maintain their physical exercise routine in their homes, even in small environments such as a balcony.

Studies show that ∼ 5% of CrossFit practitioners have some degree of addiction to exercise. 19 If this motivation may have been the explanation for maintaining their routine at home, on the other hand, it concerns the effect of high-intensity exercise on the immune system. It is observed that 22 athletes reported having symptoms of COVID-19 and, as a treatment, they did social isolation. It has been shown that high-intensity training can lead to lymphocyte apoptosis and predispose to immunosuppression. 20 Even in recreational athletes, a CrossFit training protocol with 10 repetitions of horizontal bench press, deadlifts, and squats, performed at 75% of a maximum repetition, and without a break, significantly increases interleukin-6 (IL-6). In terms of COVID-19, data suggest that IL-6 may play a key role in the evolution of the inflammatory immune response that causes acute respiratory distress syndrome. 21

The reduction of UI during exercise after starting the quarantine was statistically significant and the reasons provided by the respondents were the interruption of double under exercises and the reduction in the intensity of the training. Double under is the CrossFit movement that most frequently increases the intra-abdominal pressure (429 cm H _2_ O), with this value being one of the highest ever attributed to any sporting gesture. 5 In a previous study performed from our group, it was the exercise mostly associated with UI. 6 Thus, the interruption of this exercise in the quarantine explains the improvement in UI. Another important fact is that the increase in intra-abdominal pressure in CrossFit exercise varies with the intensity of the training, repetitions, and the use or not of load. 8 In this sense, the quarantine may have indirectly assured the protective effect to the pelvic floor, due to the difficulty in using training accessories and to the decreased training intensity.

The volume of training has been reported in previous studies as a risk factor for athletic incontinence. Women who play high-impact sports or who have a higher volume of training should be aware of the symptoms associated with pelvic floor dysfunction. 9 10 The level of exercise can be calculated based on the time (in minutes) usually spent per week or the intensity (light, moderate, intense). Normally, athletes, from any modality and who train for competitive purposes, are at twice the risk for UI when compared with sedentary individual.

Some reports indicate that physical inactivity will persist long after we recover from the COVID-19 pandemic. 13 In this sense, maintaining the CrossFit training routine at home was a positive element for future health. 22 Moreover, our findings may suggest that the general gynecologist may counsel UI women who practice CrossFit to reduce training intensity or to replace series containing jumping exercises, as this may benefit their urinary symptoms. These women may be physically active with no impact in their pelvic floor. Moreover, we may extend this by noticing that, even during a major pandemic, physical activity was not completely impaired. This experience may prepare us for future waves of this pandemic (or to others that might come).

To our knowledge, this is the first study analyzing the impact of COVID-19 quarantine on CrossFit training and the repercussion on UI during exercise. The limitations of the present study include the use of an online questionnaire, the lack of physical examination, and the self-report of COVID-19 symptoms without laboratorial confirmation. Despite sample size calculation having been performed, there is a risk of type 2 error regarding the analysis of the associated factors for the reduction of CrossFit training, and for this reason we opted to not perform a multivariate analysis.

## Conclusion

In conclusion, the COVID-19 quarantine improved athletic incontinence in CrossFit practitioners due to decreased training intensity and to interruption of higher intra-abdominal pressure exercises.
